# Characterizing the mutational burden, DNA methylation landscape, and proteome of germ cell tumor-related somatic-type malignancies to identify the tissue-of-origin, mechanisms of therapy resistance, and druggable targets

**DOI:** 10.1038/s41416-023-02425-5

**Published:** 2023-09-19

**Authors:** Felix Bremmer, Pailin Pongratanakul, Margaretha Skowron, Yue Che, Annika Richter, Stefan Küffer, Kirsten Reuter-Jessen, Hanibal Bohnenberger, Stella Pauls, Catena Kresbach, Ulrich Schüller, Kai Stühler, Philipp Ströbel, Peter Albers, Daniel Nettersheim

**Affiliations:** 1 Institute of Pathology, University Medical Center Goettingen, Goettingen, Germany; 2 Department of Urology, Urological Research Laboratory, Translational UroOncology, Medical Faculty and University Hospital Düsseldorf, Heinrich Heine University Düsseldorf, Düsseldorf, Germany; 3 Department of Urology, Medical Faculty and University Hospital Düsseldorf, Heinrich Heine University Düsseldorf, Düsseldorf, Germany; 4 Molecular Proteomics Laboratory (MPL), Biological and Medical Research Centre (BMFZ), Medical Faculty and University Hospital Düsseldorf, Heinrich Heine University Düsseldorf, Düsseldorf, Germany; 5 Institute of Neuropathology, University Hospital Hamburg-Eppendorf, Hamburg, Germany

**Keywords:** Molecular medicine, Germ cell tumours, DNA methylation, Protein-protein interaction networks

## Abstract

**Background:**

Germ cell tumors (GCT) might undergo transformation into a somatic-type malignancy (STM), resulting in a cell fate switch to tumors usually found in somatic tissues, such as rhabdomyosarcomas or adenocarcinomas. STM is associated with a poor prognosis, but the molecular and epigenetic mechanisms triggering STM are still enigmatic, the tissue-of-origin is under debate and biomarkers are lacking.

**Methods:**

To address these questions, we characterized a unique cohort of STM tissues on mutational, epigenetic and protein level using modern and high-throughput methods like TSO assays, 850k DNA methylation arrays and mass spectrometry.

**Results and conclusions:**

For the first time, we show that based on DNA methylation and proteome data carcinoma-related STM more closely resemble yolk-sac tumors, while sarcoma-related STM resemble teratoma. STM harbor mutations in FGF signaling factors (*FGF6/23, FGFR1/4)* highlighting the corresponding pathway as a therapeutic target. Furthermore, STM utilize signaling pathways, like AKT, FGF, MAPK, and WNT to mediate molecular functions coping with oxidative stress, toxin transport, DNA helicase activity, apoptosis and the cell cycle. Collectively, these data might explain the high therapy resistance of STM. Finally, we identified putative novel biomarkers secreted by STM, like EFEMP1, MIF, and DNA methylation at specific CpG dinucleotides.

## Introduction

Testicular germ cell tumors (GCT) represent a heterogeneous group with different histological subtypes stratified into seminomas and non-seminomas [1, 2]. Based on histology, gene expression profiles and epigenetics, seminomas are considered to be the default developmental pathway of the precursor lesion germ cell neoplasia in situ (GCNIS), which itself is the result of a defective primordial germ cell development. In contrast, non-seminomas arise by reprogramming of GCNIS cells to a pluripotent embryonal carcinoma (EC) [1, 2]. EC are able to differentiate into cells of all three germ layers (teratoma) or into extra-embryonic tissues (yolk-sac tumor (YST), choriocarcinoma [1–3].

A rare but deadly subtype of GCT is the somatic-type malignancy (STM), a secondary tumor component of non-seminomas that resembles cancers seen in other organs and tissues [4]. A STM is defined in the current WHO classification (5th edition) as an area of ≥ 5 mm diameter with a population of atypical mesenchymal or epithelial cells [5]. These STM span a wide variety of tumors, including rhabdomyosarcomas, adenocarcinomas, and embryonic-type neuroectodermal tumors (ENET). STM occur with an incidence of 2–6% at any point of GCT development, but are mainly diagnosed at a metastatic stage in a post-chemotherapeutic setting [6]. Patients with STM face a poor prognosis with a 5-year survival rate of 50–60% due to resistance towards cisplatin-based chemotherapy [7, 8]. Unfortunately, treatment guidelines are still missing due to a lack of knowledge about this special group of cancers and their biology.

Most GCT-related STM are found in association with TER, leading to the assumption that TER is the tissue-of-origin [9–12]. Nevertheless, there are also STM occurring in GCT without TER and in association with YST, indicating that YST cells (in particular their mesenchymal component) might transform into STM as well [13–15].

So far, the developmental origin and the underlying molecular and (epi)genetic mechanisms of STM formation remain elusive. Since specific treatments are still lacking, further research on the origin and pathogenesis of STM and the identification of potential therapeutic targets, is warranted. Thus, this study characterized the molecular and (epi)genetic features of STM on mutational, DNA methylation, and proteome level to identify the key processes driving STM formation and related therapy resistance, the tissue-of-origin as well as new therapeutic options and novel biomarkers.

## Material and methods

### GCT/STM tissues

All GCT/STM tissues included in this study were collected from local biobanks (Institutes of Pathology at University Hospital Düsseldorf and University Medical Center Göttingen). All samples were re-evaluated by a reference pathologist for type II GCT (F.B.). In this study, we analyzed a GCT-related STM cohort consisting of 13 adenocarcinomas, 7 rhabdomyosarcomas, 4 carcinomas not otherwise specified (NOS), 2 angiosarcomas, 2 sarcomas without lineage-specific differentiation, and 2 ENET (*n* = 30 in total) (Data S1A). We included 10 TER and 5 YST without STM as controls (Data S1A). The diagnosis had been made according to the WHO criteria of STM [5]. The STM accompanying histology is also given in “Data S1A”.

### Immunohistochemistry

Immunohistochemistry (IHC) has been performed as described earlier [15]. Briefly, antigen retrieval was carried out in citrate-buffer. The primary antibodies were incubated for 30 min (min) at room temperature (RT). Sections were incubated with a ready-to-use-HRP-labeled secondary antibody at RT for 25 min. The substrate DAB+ Chromogen system was used to visualize the antigen. Tissues were counterstained with Meyer’s hematoxylin. An overview of all IHC results is given in Data S1A (Data S1A). In total, 26 samples were analyzed (10 adenocarcinoma, 3 carcinoma NOS, 8 rhabdomyosarcoma, 2 angiosarcoma, 1 sarcoma, 2 ENET).

### Nucleic acid isolation

The STM area was highlighted on H&E-stained slides prior to the analysis by a reference pathologist for GCT. Only the marked areas were isolated from the FFPE-slides. DNA was extracted from 2×5 µm FFPE slices using the InnuPREP FFPE DNA Kit on the InnuPure C16 System (Jena Analytika, Jena, Germany) according to manufacturer instructions. RNA was isolated from 2×5 µm slices using the Maxwell RNA extraction kit (Promega, Walldorf, Germany) according to manufacturer’s recommendations. DNA and RNA concentrations were measured on the Qubit 3 Fluorometer (ThermoScientific, Paisley, UK).

### 12p gain PCR analysis

A PCR analysis measuring the 12p gain status of STM tissues was performed exactly as published [16]. A fold change normalized to controls of > 2 was set as a cut-off value for samples considered to harbor a 12p gain. In total, ten samples were analyzed (two adenocarcinoma, two carcinoma NOS, two rhabdomyosarcoma, one angiosarcoma, one sarcoma).

### Illumina TruSight Oncology 500 (TSO) analyses

DNA libraries were prepared using the hybrid capture-based TSO Library Preparation Kit (Illumina, San Diego, CA, USA) following the manufacturer’s instructions (#1000000067621 v00). Library concentrations and peak heights were evaluated on a Tape Station (Agilent, Santa Clara, USA). Equal amounts of up to eight library samples were pooled and diluted to 4 nM. 10 µl of the library pool was mixed in 0.1 M NaOH and incubated for 5 min at RT. The library was neutralized and diluted to 20 pMwith 990 µl HT1, mixed and kept on ice. To generate 200,000 clusters/mm^2^ the pool was diluted to 0.6 pM by the addition of 1261 µl HT1, 39 µl library (20 pM) and 1 µl PhiX (20 pM). Libraries were sequenced on an Illumina NextSeq 500 instrument. The FastQ files were analyzed in CLC Biomedical Workbench (Qiagen). Reads were mapped to hg19 followed by initial variant calling. Then local realignments, primer clipping, and low-frequency variant calling were performed. False-positives were removed based on read quality and forward/reverse balance. All variants were checked manually for sequencing artefacts. The average coverage was > 500 in all samples; the mutations had at least 50 variant reads. In total, 11 samples were analyzed (2 adenocarcinoma, 2 carcinoma NOS, 3 rhabdomyosarcoma, 1 angiosarcoma, 2 sarcoma).

### Illumina 850k DNA methylation array (850k array)

DNA was isolated from FFPE tissue using the ReliaPrep™ FFPE gDNA Miniprep System (Promega, Walldorf, Germany) according to manufacturer’s instructions. 100–500 ng DNA were used for bisulfite conversion with the EZ DNA Methylation Kit (Zymo Research, Freiburg. i. B., Germany). Afterwards, the DNA Clean & Concentrator-5 (Zymo Research) and the Infinium HD FFPE DNA Restore Kit (Illumina) were used to clean and restore the converted DNA. Finally, the Infinium MethylationEPIC BeadChip (Illumina) was used to evaluate the methylation status of 850,000 CpG sites on an iScan device (Illumina). In total, 11 samples were analyzed (5 adenocarcinoma, 6 rhabdomyosarcoma, 5 TER, 4 YST).

### Liquid chromatography coupled to mass spectrometry (LC-MS)

For sample preparation, a modified FFPE tissue lysis protocol of Ikeda et al. was applied [17]. FFPE tissues were deparaffinized by shaking in 500 µL Xylene for 5 min, followed by removal of the solvent and air-dry the residual solvent. Tissues were resuspended in 200 µL lysis buffer (300 mM TRIS/HCl, 2% SDS, pH 8.0), shock-frozen in liquid nitrogen and immediately heated for 25 min at 99 °C and 350 rounds per minute (rpm). Samples were ultrasonicated on ice for 20 min with 30 seconds (s) on/off cycles and then shook for 2 hours (h) at 80 °C and 500 rpm followed by a second ultrasonication step. After centrifugation for 5 min at 3500 rpm, the pellet was resuspended in 100 µL lysis buffer for a second extraction round. Supernatants were combined and protein concentration was determined using the Pierce 660 nm Protein Assay (Thermo Fisher Scientific, Idstein, Germany).

For LC-MS analysis a modified magnetic bead-based sample preparation protocol according to Hughes and colleagues were applied [18]. Briefly, 20 µg total protein were reduced by adding 10 µL 300 mM DTT and shaking for 20 min at 56 °C and 1000 rpm, followed by alkylation with the addition of 13 µL 100 mM IAA and incubation for 15 min in the dark. 10 µl of a 20 µg/µl bead stock (1:1 Sera-Mag SpeedBeads) were added to each sample. For protein aggregation capture, ethanol (EtOH) was added to a final concentration of 80% and incubated for 15 min at 20 °C. After three rinsing steps with 80% EtOH and one rinsing step with 100% ACN, beads were resuspended in 50 mM TEAB buffer and digested with final 1:50 trypsin at 37 °C and 1000 rpm overnight. Extra-digestion was carried out by adding trypsin (final 1:50) and shaking at 37 °C and 1000 rpm for 4 h. 500 ng of each sample were subjected to LC-MS.

For the LC-MS acquisition an Orbitrap Fusion Lumos Tribrid Mass Spectrometer coupled to an Ultimate 3000 Rapid Separation liquid chromatography system equipped with an Acclaim PepMap 100 C18 column (75 µm inner diameter, 25 cm length, 2 mm particle size) as separation column and an Acclaim PepMap 100 C18 column (75 µm inner diameter, 2 cm length, 2 mm particle size) as trap column (all equipment from Thermo Fisher Scientific). A LC-gradient of 180 min was applied and the MS operated in positive mode with a scan range of 200–2000 *m*/*z* at a resolution of 120,000. The capillary temperature was set to 275 °C, the source voltage (V) to 1.5 kV, the normalized AGC target was set to 62.5% and the maximum injection time was 60 ms. HCD fragmentations were carried out within a cycle time of 2 s.

Data were analyzed by Proteome Discoverer (version 2.4.1.15, Thermo Fisher Scientific). RAW files were matched against the human Swissprot database (Download: 23.01.2020) and the Maxquant Contaminant database (Download: 20.02.2021), using SequestHT integrated in the LFQ Tribrid processing workflow (Thermo Fisher Scientific). The maximum number of missed cleavages was set to 2 and the peptide length was 6–144 amino acids. Precursor mass tolerance was set to 10 ppm and the fragment mass tolerance was 0.6 Dalton. All samples were analyzed in a match between run search. Post processing, peptides were ungrouped and filtered to 1% FDR on protein and peptide level and to all proteins identified with ≥ 2 peptides. Contaminants were filtered out.

In total, 46 samples were analyzed (7 adenocarcinoma, 5 carcinoma NOS, 10 rhabdomyosarcoma, 5 angiosarcoma, 2 sarcoma, 2 ENET, 10 TER, 5 YST).

### Online analyses tools and software

“The Cancer Genome Atlas” (TCGA) datasets were analyzed using “cBioPortal for Cancer Genomics” [19]. STRING was used to predict protein–protein-interaction by confidence [20]. DAVID was used to predict molecular and biological functions of proteins based on “Gene Ontology” (GO) [21]. “Phyton” was used to generate volcano and violin plots [22, 23]. Venn diagrams were generated by “Venny 2.1.0” [24]. Pearson’s correlation matrixes and heatmaps were generated by “Morpheus” (https://software.broadinstitute.org/Morpheus).

## Results

**Fig. 1:**
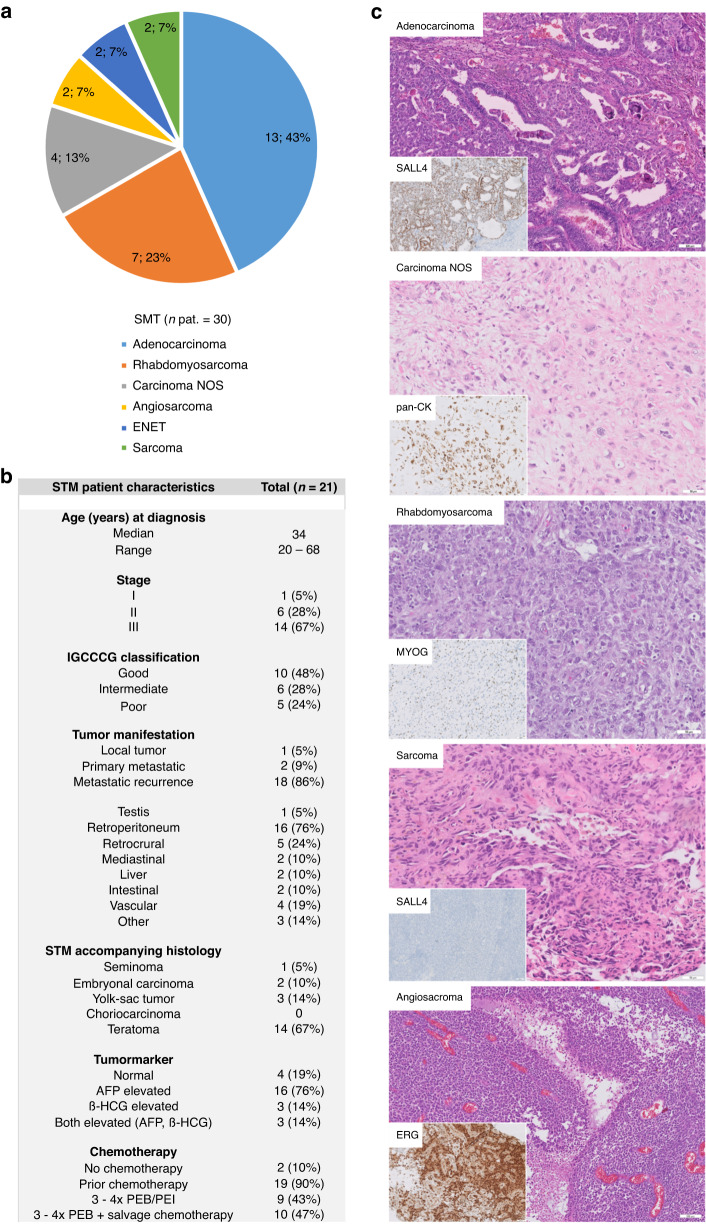
Clinical and histological features of the STM cohort. **a** Pie chart summarizing distribution of the various STM entities analyzed in this study. **b** Clinical parameters of the STM cohort (at diagnosis of STM) from the University Hospital Düsseldorf (Department of Urology) analyzed in this study. **c** Exemplary H&E stainings of each STM entity and IHC staining of typical marker proteins.

In this study, we analyzed the molecular and (epi)genetic features of a cohort of GCT-related STM consisting of 13 adenocarcinomas, 7 rhabdomyosarcomas, 4 carcinomas not otherwise specified (NOS), 2 angiosarcomas, 2 sarcomas without lineage-specific differentiation, and 2 ENET (Fig. 1a) (*n* = 30 in total). We included 10 TER and 5 YST without STM as controls (Data S1A).

Detailed clinical data were available from 21 STM patients. Ages ranged from 20 to 68 years (mean 34). The majority of patients (20/21; 95%) who developed a STM initially presented with metastasis with 5% in CS I (1), 28% in CS II (6), and 67% in CS III (14) (Fig. 1a). Prognosis of the IGCCCG risk classification was mainly favorable with good, intermediate and poor prognosis in 48% (10), 28% (6), and 24% (5) of patients, respectively (Fig. 1b). Prior to the diagnosis of STM, 90% of patients had received at least three cycles of platin-based chemotherapy (Fig. 1b). 2 (10%) patients presented with a STM at first diagnosis. At the time of STM diagnosis, 76% (16) and 14% (3) of patients had elevated AFP (> 7 µg/l) or ß-hCG (> 2 mU/ml), respectively (Fig. 1b). ß-hCG was only elevated in combination with AFP (Fig. 1b). Normal tumor markers were found in 19% (4) (Fig. 1a; Data S1A). STM mainly manifested at retroperitoneal lymph nodes and retrocrural sites (Fig. 1b). Exemplary H&E stainings and IHC of typical markers for each STM entity are given in “Fig. 1c”, while an overview of all performed IHC stainings is given in “Data S1A”. Histologically, the adenocarcinomas were composed of neoplastic glands with nuclear atypia. In IHC, all cases were SALL4^+^, focally FOXA2^+^ and CDX2^+^ as well as AFP^−^, GPC3^−^, CK7^−^ and TTF-1^−^ in most cases. The proliferation rate (Ki67) was between 30 and 50%. The rhabdomysarcomas were composed of spindled rhabdoid cells with pleomorphic nuclei. The IHC detected Myogenin^+^ and Desmin^+^ cells. The tumor cells were SALL4^-^, Caldesmon^−^ and Actin^-^. The carcinomas NOS contained highly atypical cells with pleomorphic nuclei and without any noticeable pattern. The IHC detected pan-cytokeratin^+^ and SALL4^−^ cells. The proliferation rate was between 20 and 25%. The sarcoma NOS cells were completely pan-cytokeratin^-^ with focal Actin^+^ cells (without any noticeable pattern). The pleomorphic tumor cells showed a high proliferation rate (> 50%). The angiosarcomas showed anastomosing vascular spaces lined with atypical cells. Other parts showed a solid architecture with epithelioid or spindled cells. The IHC revealed CD31^+^, ERG^+^, CD34^−^, and SALL4^−^ cells. The ENET samples contained small cells with minimal to modest pale eosinophilic cytoplasm and round to oval hyperchromatic nuclei. In IHC, the cells presented as pan-cytokeratin^+^, S100^−^, chromogranin^−^, and scattered synaptophysin^+^. The YST showed a variety of patterns composed of neoplastic glands with prismatic cells. The IHC detected SALL4^+^, FOXA2^+^, GPC3^+^, and AFP^+^ cells. For TER, a typical arrangement of cells of all three germ layers (ectoderm, mesoderm, endoderm) was observed.

**Fig. 2:**
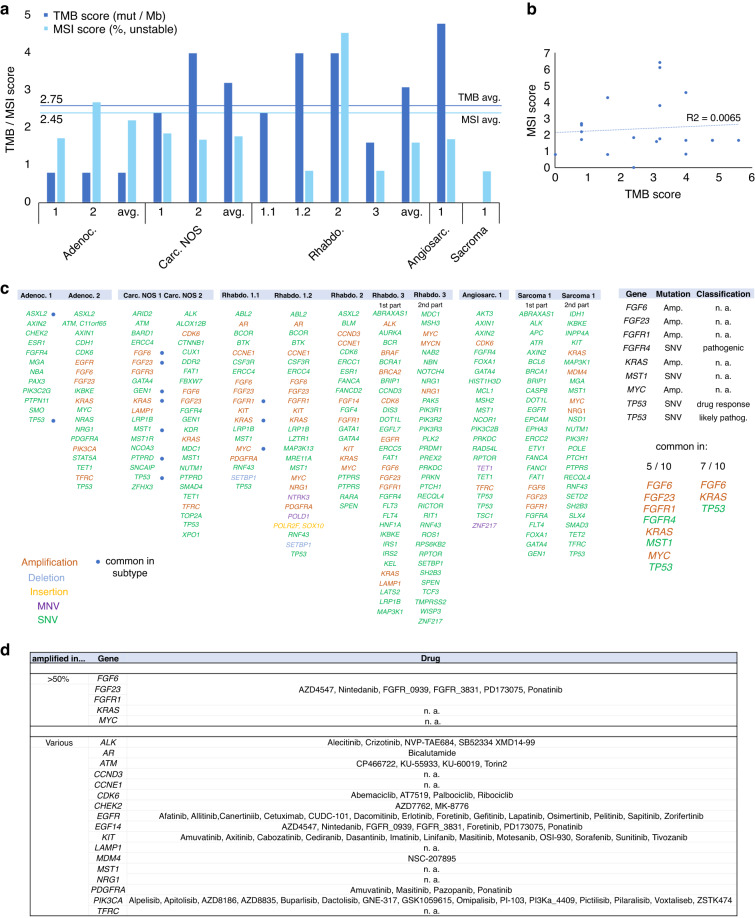
Analyzing druggable mutations of STM tissues. **a**, **b** Illustration of the tumor mutational burden (TMB; mutations/megabase) and microsatellite instability score (MSI; % unstable) (**a**) and the ratio of both parameters (**b**) in STM samples analyzed by the TSO assay. **c** All detected mutations in indicated STM samples. Blue dots label mutations found in all samples of a STM subgroup. MNV: multiple nucleotide variants; SNV: single nucleotide variants. **d** Overview of drugs targeting found amplified genes/signaling factors.

By a TSO analysis, we analyzed the mutational burden of the STM samples. We included 2 adenocarcinomas, 2 carcinomas NOS, 4 rhabdomyosarcomas, 1 angiosarcoma and 1 sarcoma (in total *n* = 10). The rhabdomyosarcomas included a primary tumor (Rhabdo. 1.1) and a metastasis (Rhabdo. 1.2) of the same patient. All STM samples harbored the GCT-typical chromosome 12p gain, confirming their GCT origin (Fig. S1A) [16]. As expected for a GCT-derived malignancy, the tumor mutational burden (TMB) score (avg. 2.75 mutations/megabase) and microsatellite instability (MSI) score (avg. 2.45% unstable sites) were quite low in all STM (Fig. 2a). No correlation between TMB and MSI score was found (Fig. 2b). An overview of all detected genetic variants is given in “Data S1B, C”. All mutations classified as “(likely) benign” or known to be commonly distributed in the human population without any effect were excluded from further analyses. In adenocarcinomas, common alterations included *ASXL2* and *TP53*. In carcinomas NOS, frequent mutations included *FGF23*, *FGF6*, *GEN1*, *KRAS*, *MST1*, *PTPRD*, and *TP53* (Fig. 2c; Data S1B). Among the rhabdomyosarcomas, alterations in *FGFR1*, *KRAS*, and *MYC* were detected (Fig. 2c; Data S1B). Taking all STM samples together, *FGF6*, *KRAS*, and *TP53* were mutated in at least 70% of samples, while additionally *FGF23, FGFR1, FGFR4, MST1*, and *MYC* were mutated in at least 50% (Fig. 2c). SNP-mutations affecting *FGFR4* (c.1162G>A) and *TP53* (c.215C>G; c.380C>T) were classified as “pathogenic” or as affecting “drug response”, respectively, highlighting these mutated factors as putative therapeutic targets (Fig. 2c). We summarized drugs known to target the factors affected by mutations in “Fig. 2d”.

We next compared the mutational status of all genes found mutated by our TSO screen in any of the STM samples to the TCGA GCT cohort (149 samples) (Fig. S1B). *KRAS* (STM: > 70%; GCT: 16%) and *KIT* (STM: 30%; GCT: 14%) were the most amplified or missense mutated genes in the GCT cohort with the majority being mutated in seminomas (Fig. S1B). *FGF6* (STM: > 70%; GCT: 5%) and *FGF23* (STM: > 70%; GCT: 5%) were amplified in the same eight GCT samples (mainly non-seminomas) (Fig. S1B). Thus, *FGF6* and *FGF23* were also amplified in GCT, but with a considerably lower frequency than in STM. All other questioned genes were mutated with very low frequency (mainly < 1%) in GCT samples (Fig. S1B).

**Fig. 3:**
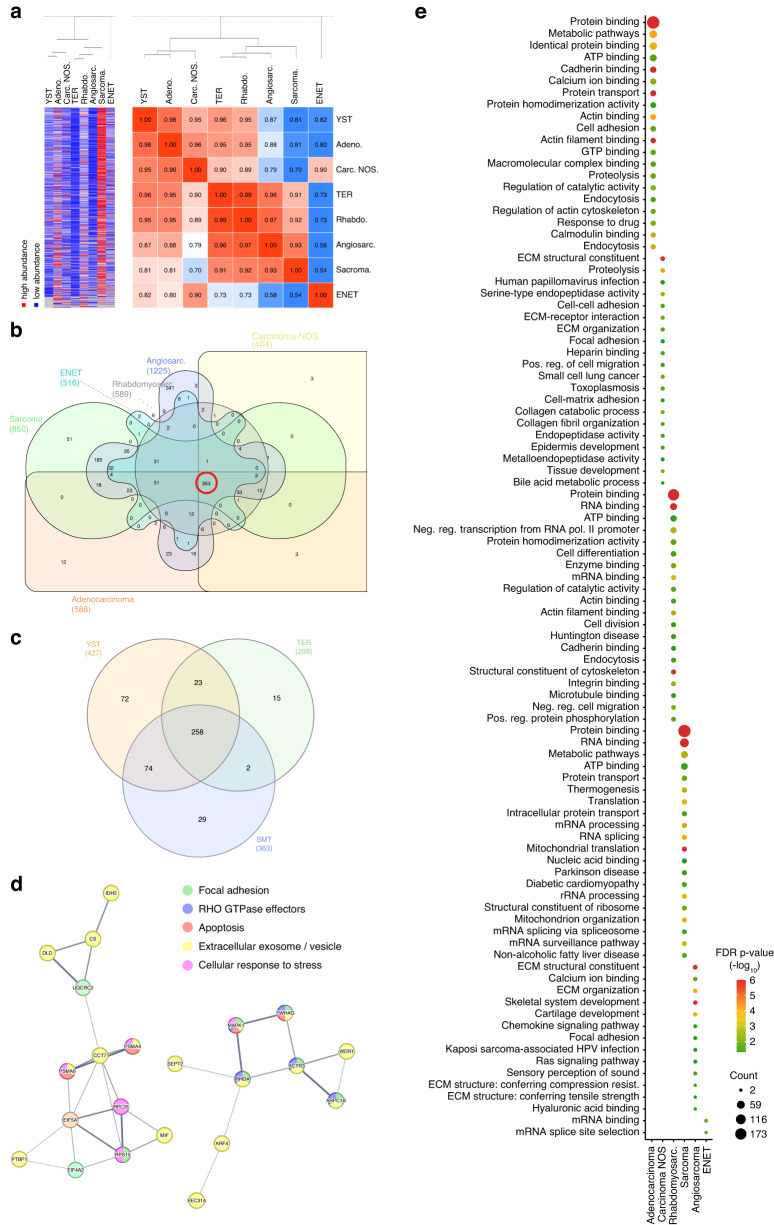
Analyzing the proteome of STM tissues. **a** A heatmap including hierarchical clustering and a Pearson’s correlation matrix illustrate similarities and differences in the proteome (abundance > 10^7^) between the various STM groups as well as YST and TER. By Venn diagrams, shared and unique proteins (abundance > 10^7^) were identified between the STM entities (**b**) and compared to YST/TER (**c**). 363 proteins were found in all analyzed STM entities (**b**, red circle). **d** STRING-based protein-protein-interaction prediction of proteins commonly found in STM entities, but not in TER or YST. **e** DAVID-based GO and KEGG screen for biological processes and functions related to the proteins found exclusively in each STM entity.

By using LC-MS, we analyzed the proteome of STM (adenocarcinomas (*n* = 7), carcinomas NOS (*n* = 5), angiosarcomas (*n* = 5), ENET (*n* = 2), rhabdomyosarcomas (*n* = 11), sarcomas NOS (*n* = 3)). YST (*n* = 9) and TER (*n* = 20) served as controls. All samples showed comparable abundance levels of detected proteins (in total 3025) (Fig. S1C; Data S1D). As described initially, both TER and YST are believed to be the origin of STM [9–15]. To address this question, we compared STM to YST and TER samples by hierarchical clustering and in a Pearson’s correlation matrix (PCM) (Fig. 3a). Here, adenocarcinoma and carcinoma NOS (carcinoma-related entities) clustered to YST, while rhabdomyosarcomas, sarcomas and angiosarcomas (sarcoma-related entities) clustered to TER, (Fig. 3a). ENET cases were considerably different from the other entities, but were more similar to YST than TER (Fig. 3a).

By comparing the proteomes between STM and YST/TER using volcano plots, we highlighted the top 10 significantly enriched or depleted proteins (Fig. S2). We identified 363 common proteins (abundance > 10^6^) between all STM samples (Fig. 3b; Data S1E). We screened for putative interactions of these proteins and enriched GO categories using STRING (Fig. S3A, B). The proteins were predicted to be involved in translational initiation, regulation of cell death, the extracellular matrix (ECM), secretion, cell differentiation and the immune system (Fig. S3A). Functional clustering using DAVID demonstrated involvement of these proteins in toxin transport and telomere maintenance (cluster 4), positive regulation of signal transduction by p53 (6), antioxidant activity and removal of oxide radicals (clusters 8, 11), response to stress (8), DNA binding and regulation of DNA recombination (cluster 12), DNA helicase activity and duplex unwinding (cluster 35), chromatin remodeling (clusters 12, 20), regulation of apoptosis (clusters 19, 34), and the cell cycle (cluster 30) (Data S1E). Additionally, ECM-associated processes like ECM organization and collagen binding as well as adhesion and migration (clusters 19, 26, 29) were enriched (Data S1E). Furthermore, processes linked to activation of the (adaptive and innate) immune system, complement activation, Ig binding, neutrophil chemotaxis and B cell activation/signaling were enriched (clusters 21, 24, 25, 33) (Data S1E). To mediate these processes, interleukin, MAPK, WNT, FGFR2, PI3K-AKT, NOTCH, and HIF-1 signaling seem to be utilized via Rho GTPase effectors and ECM receptors (Fig. S3B). Furthermore, epigenetic processes like histone deacetylase binding, nucleosome positioning and chromatin remodeling (clusters 9, 21) were enriched, suggesting that development of STM is accompanied by epigenetic alterations (clusters 12, 17, 20) (Data S1E).

Comparing all proteins commonly found in STM entities to proteins found in TER and YST, an overlap of 54.5% (258 proteins) was found, while 29 proteins were exclusively found in STM (Fig. 3c). 22 of these 29 (75.8%) proteins were predicted to interact with each other, and were mainly related to focal adhesion, extracellular exosomes/vesicles, apoptosis, cellular response to stress, RHO GTPase effectors, and MAPK signaling (Fig. 3d). Two proteins, EFEMP1 and MIF are extracellular factors or cytokines secreted exclusively by the STM, highlighting these proteins as putative biomarkers (Data S1E).

Next, we searched for unique features of each STM entity by the DAVID algorithm (Fig. 3e; Data S1L). Proteins found exclusively in adenocarcinomas were linked to cell adhesion and migration, cadherin binding, endocytosis, response to drug and hypoxia, oxidoreductase activity, and regulation of angiogenesis (Fig. 3e). In carcinomas NOS, processes mainly related to the ECM (structure, organization, receptor-interaction, collagen catabolic process/fibril organization, metalloendopeptidase activity), adhesion, and migration were found (Fig. 3e). In rhabdomyosarcomas, unique proteins were associated with endocytosis, cadherin and integrin binding, cell differentiation (multicellular organism development, cardiac muscle contraction, response to TGF-β stimulus), and cell division (Fig. 3e). In sarcomas NOS, unique proteins were linked to regulation of NFkB signaling, RNA regulation (splicing, rRNA processing, mRNA surveillance, spliceosome), and protein biosynthesis and trafficking (ribosome structure, protein transport, mitochondrial translation, ribosome biogenesis) (Fig. 3e). In angiosarcomas, processes related to the ECM (organization, structure, compression resistance, tensile strength, hyaluronic acid and heparin binding), adhesion, chemokine signaling pathways (RAS signaling), and mesodermal differentiation (skeletal system development, cartilage development) were found (Fig. 3e). Taken together, several key molecular functions are shared between STM (ECM interaction, molecule trafficking, adhesion, migration), although each entity engages different proteins to realize these functions.

**Fig. 4:**
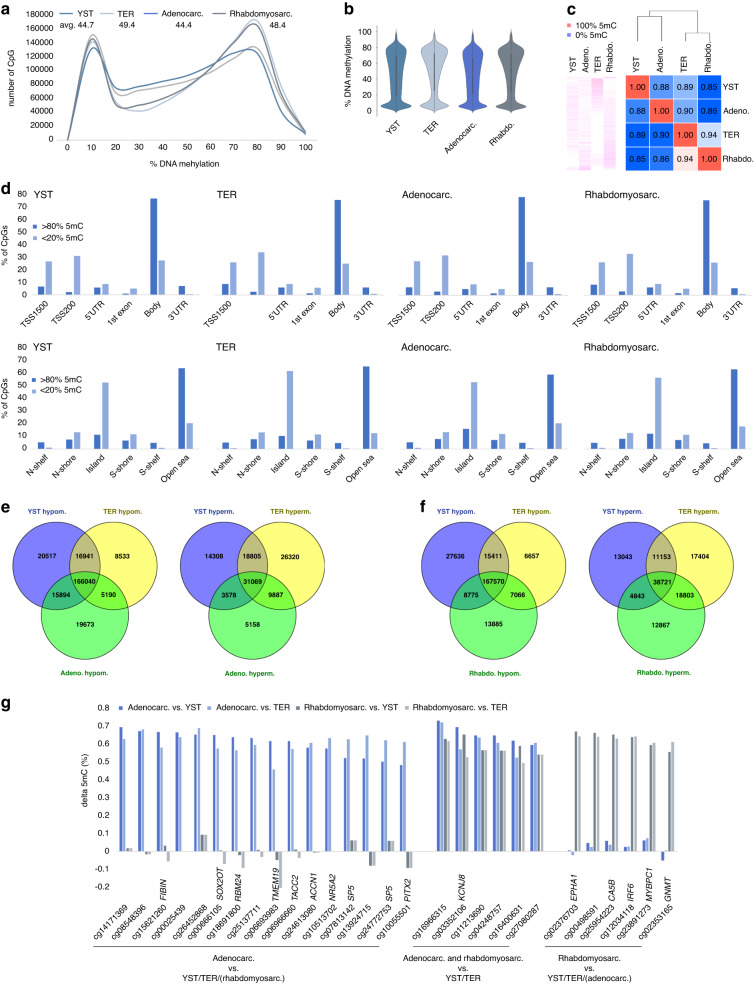
Analyzing the DNA methylation profile of STM tissues. **a** Distribution of DNA methylation levels (%) across all analyzed CpG dinucleotides. **b** A violin plots illustrates genome-wide distribution of DNA methylation levels. **C** A heatmap and a Pearson’s correlation matrix including hierarchical clustering illustrates and compares DNA methylation data, respectively. **d** Distribution of DNA hypo- (< 20%) and hypermethylated (> 80%) CpG dinucleotides across genomic regions/CpG islands. Venn diagrams comparing hyper- and hypomethylated CpG dinucleotides in adenocarcinomas (**e**) and rhabdomyosarcomas (**f**) with YST and TER. **g** Putative epigenetic biomarkers for adenocarcinomas and/or rhabdomyosarcomas based on the DNA methylation status of single CpG dinucleotides.

To analyze differences in the DNA methylation (5mC) landscape, we performed Illumina 850k DNA methylation arrays. We included the two most common STM subtypes (i.e., adenocarcinomas (*n* = 5) and rhabdomyosarcoma (*n* = 5)), while YST (*n* = 5) and TER (*n* = 5) served as controls (Data S1F). On a global level, compared to YST and adenocarcinomas, TER and rhabdomyosarcomas showed a higher amount of hypermethylated (>80%) sites, while YST and adenocarcinomas presented with a higher proportion of CpG dinucleotides with intermediate (20–80%) 5 mC levels (Fig. 4a, b). The average 5mC levels were similar between TER and rhabdomyosarcomas (49.4 and 48.4%), followed by YST and adenocarcinomas with slightly lower levels (44.7 and 44.4%) (Fig. 4a). By performing hierarchical clustering and a PCM, we demonstrated that YST and adenocarcinomas grouped to each other, while TER grouped with rhabdomyosarcomas (Fig. 4c). When sorting the 5mC data for regions showing only hypo- (< 20%) or hypermethylation (> 80%), followed by screening for distribution across genomic regions/CpG islands, we found that hypermethylated regions where strongly associated with gene bodies (i.e., coding regions) and open sea (i.e., not in CpG island context), while hypomethylated regions where mainly found at transcription start sites (TSS200, TSS1500) and in CpG island context (Fig. 4d). No considerable differences regarding 5mC distribution were observed between STM and YST/TER (Fig. 4d). We compared all CpG dinucleotides found hypo- (< 20%) or hypermethylated (> 80%) in adenocarcinomas or rhabdomyosarcomas to the CpG dinucleotides identified in YST or TER (Fig. 4e, f). Here, a considerable overlap of hypomethylated CpG dinucleotides was found between adenocarcinomas and YST, while in rhabdomyosarcomas a big proportion of hypermethylated CpG overlapped with TER, again reflecting the different 5mC distributions between adenocarcinomas/YST and rhabdomyosarcomas/TER (Figs. 4e, f; 1a, b).

By volcano plots, we identified differentially methylated CpG dinucleotides between adenocarcinomas and rhabdomyosarcomas compared to YST/TER (Fig. S4; Data S1G, H). We grouped the identified CpG dinucleotides (initial Δ5mC > 60%) for their ability to discriminate a given STM from the other types (Fig. 4g). These hypermethylated CpG dinucleotides might serve as epigenetic biomarkers to detect the occurrence of STM, e.g., by screening cell-free DNA.

Additionally, we correlated 5mC data (with hypo- (< 20%) or hypermethylation (> 80%) in at least 60% of all questioned CpG dinucleotides linked to an annotated gene) to the proteome data (abundance > 10^6^) (Data S1I). In adenocarcinomas, we found 418 hypomethylated genes correlating to protein production and 384 genes/proteins in rhabdomyosarcomas, of which 326 were found shared between both STM entities (Fig. S4B; Data S1I). We found no hypermethylated genes correlating to protein production (Fig. S4B; Data S1I).

## Discussion

In this study, we characterized various GCT-related STM subtypes at the mutational, DNA methylation and proteome level and compared them to YST and TER.

The overall mutational burden including amplification fold changes were GCT typically low in STM, suggesting that mutations are not a crucial driver of STM formation. Nevertheless, our data and the correlation to the TCGA GCT cohort suggest that mutations detected in STM arose during formation of STM and are not generally detectable in GCT. We found amplifications in oncogenes, like *KRAS* or *MYC*, or mutations in *TP53*, which might affect drug response (c.215C>G), as well as FGF signaling factors might contribute to the aggressive character of STM by triggering proliferation, survival and anti-apoptotic signals. With mutations found in *FGF6*, *FGF23*, *FGFR1,* and *FGFR4* in at least 50% of the samples, FGF signaling seems to be a priority target of mutational events. There are some drugs available, mainly small molecule inhibitors and receptor-tyrosine-kinase inhibitors, targeting the FGF signaling cascade, i.e., AZD4547 (targeting FGFR1-3, but not FGFR4), Nintendanib, FGFR_0939, FGFR_3821, PD173075 and Ponatinib. Several completed or ongoing clinical trials screening some of these drugs were found (clinicaltrials.gov); AZD4547: 12, Nintendanib: 164, Ponatinib: 60. So, several FGF signaling related therapeutic options for treatment of STM are available and should be screened in follow-up studies and eventually clinical trials. Of note, although mutations in *KRAS*, *MYC,* and *TP53* were detected, to date no drugs targeting the specific mutations found in this study are available.

**Fig. 5:**
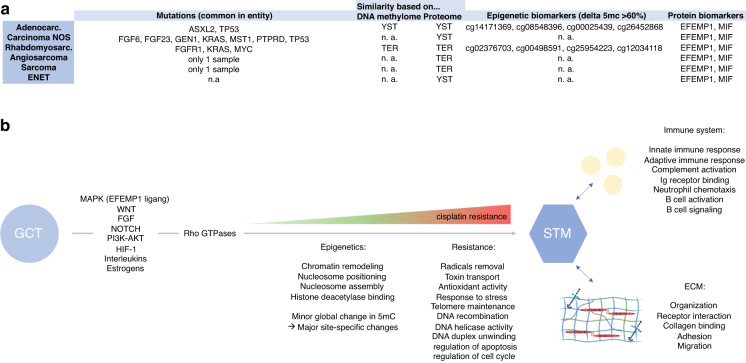
Key findings of this study. **a** Summary of found mutations common in each STM entity as well as of similarities of STM entities to YST/TER on DNA methylation and proteome level. **b** Summary of molecular and epigenetic processes commonly found in STM entities putatively mediating therapy resistance and interaction with cells of the immune system and the ECM. Parts of this figure were generated by biorender.com.

As found by LC-MS, the STM entities commonly utilize MAPK, WNT, FGF, NOTCH, PI3K-AKT, and HIF-1 signaling to mediate processes like response to oxidative stress, toxin transport, oxidant detoxification, DNA helicase activity, DNA duplex unwinding, the cell cycle and apoptosis (Fig. 5b). In combination with the frequently found SNV in *TP53* (c.215C>G), which might affect drug response, these processes might contribute to the insensitivity of the STM entities towards the cisplatin-based therapy by affecting key steps of cisplatin turnover, like influx/efflux, DNA repair, formation of radicals and (oxidative) stress caused by the therapy. Furthermore, ECM- and immune system-related processes were considerably enriched in all STM, pointing at a close interaction with the surrounding microenvironment including immune cells (Fig. 5b). Some proteins mediating the related biological functions were also found in YST and TER, suggesting that these GCT entities, which are also known for their high insensitivity towards cisplatin, might utilize similar mechanisms as the STM to increase the insensitivity towards cisplatin. Nevertheless, 29 proteins involved in regulation of apoptosis, stress response and adhesion as well as extracellular secretion were exclusively found in STM. Additionally, MAPK signaling related molecules (MAPK1, 14-3-3-gamma, RhoA) and the EGF ligand EFEMP1, which has been shown to activate MAPK signaling in pancreatic adenocarcinomas, were enriched in STM compared to YST/TER [25]. Thus, in STM these proteins and MAPK signaling triggering survival and growth might further contribute to cisplatin resistance.

Our study also highlighted putative biomarkers for SMT. With EFEMP1 and MIF, we identified two proteins exclusively secreted by the STM, which might serve as liquid biomarkers of STM, e.g., by blood sample screening in GCT patients (Fig. 5b). Additionally, we identified several hypermethylated CpG dinucleotides, which might serve as epigenetic biomarkers to detect the occurrence of STM, e.g., by screening cell-free DNA (Fig. 5b).

Regarding the tissue-of-origin of STM, based on the proteome adenocarcinoma, carcinoma NOS and ENET were more similar to YST, while rhabdomyosarcomas, angiosarcomas and sarcomas NOS were more closely related to TER (Fig. 5a). Taking the DNA methylation pattern into account, again a similarity between adenocarcinomas and YST as well as rhabdomyosarcomas and TER was demonstrated (Fig. 5a). The clinical data related to our cohort showed that YST (14%) and TER (67%) were the prevalent STM accompanying histology and in 76% of all cases elevated AFP levels were detected. These data support the hypothesis that both, YST and TER, are tissues-of-origin for the various STM entities. 90% of patients received at least three cycles of chemotherapy before diagnosis of a STM, suggesting that formation of a STM represents a therapy escape mechanisms for YST/TER cells. Importantly, formation of YST and TER seems to be an escape mechanism itself, since mostly YST and TER remain after chemotherapy regimen and are the leading cause of GCT-related death. Thus, the development of YST or TER from EC under therapy and eventually a STM represents an escalating cascade of escape mechanisms for GCT cells enabling survival.

During the submission/revision process of this article, Wyvekens et al. molecularly and epigenetically characterized a STM cohort of 36 male patients [26]. There, the authors found mutations in *KRAS* and *TP53* in 28% of cases each, which is in line with our findings in the mutational screen [26]. Similar to our TSO analysis, Wyvekens et al. found no oncogenic gene fusions in nine patient samples. Regarding DNA methylation, Wyvekens et al. detected distinct DNA methylation patterns for STM (ENET and rhabdomyosarcoma) and GCT samples, which is again in line with our 850k array analysis.

### Summary and outlook

Together with the article published by Wyvekens et al., both studies shed light on the molecular and (epi)genetic features of STM in a unique cohort of patient material providing comprehensive mutation, proteome and DNA methylation data as starting point for future studies. For the first time, we show that on a molecular level carcinoma-related STM more closely resemble YST, while sarcoma-related STM resemble TER. Additionally, we identified common mutations as well as molecular and epigenetic mechanisms contributing to the therapy resistance of STM. Finally, we identified new STM biomarkers and therapeutic options to treat STM patients, which should be translated into clinical testing.

### Limitations

Limitations of this study are the relatively small number of samples analyzed for epigenetic and genomic changes, which is due to the rarity of the STM. Nevertheless, in general our cohort represents one of the largest cohorts analyzed in the field, but studying more STM cases to confirm and verify our data would be of benefit. Additionally, a molecular and epigenetic similarity between tumor types does not necessarily indicate definitive evolution from a precursor tumor subtype. Further, our cohort lacks the primary tumors of each STM patient, which would be an important control to recapitulate tumor evolution and STM formation with regard to mutations, epigenetics and changes on protein level. STM are not part of the TCGA GCT cohort, thus, comparing our findings to TCGA data is only possible for GCT in non-STM context. Furthermore, there is a lack of appropriate GCT-related STM model systems, i.e., cell lines are not available and setting up ex vivo cultures of these rarely occurring STM might be very time challenging and quite hard to organize. Thus, functional experiments or in vitro drug screenings are limited or not possible, respectively. Additionally, although we identified several drugs putatively suitable to target STM, setting up clinical trials is also very challenging due to the rarity of the STM phenomenon.

### Supplementary information

Fig. S1

Fig. S2

Fig. S3

Fig. S4

Data S1

Supplemental figure and data legends

## Data Availability

The datasets and computer code produced in this study are available in the following databases: 850k DNA methylation data: Gene expression omnibus (GSE219033); LC-MS data: ProteomeXchange (PXD039546).

## References

[1] Cheng L, Albers P, Berney DM, Feldman DR, Daugaard G, Gilligan T (2018). Testicular cancer. Nat Rev Dis Prim.

[2] Oosterhuis JW, Looijenga LHJ (2005). Testicular germ-cell tumours in a broader perspective. Nat Rev Cancer.

[3] Müller MR, Skowron MA, Albers P, Nettersheim D (2020). Molecular and epigenetic pathogenesis of germ cell tumors. Asian J Urol.

[4] Hwang MJ, Hamza A, Zhang M, Tu SM, Pisters LL, Czerniak B (2022). Somatic-type malignancies in testicular germ cell tumors: a clinicopathologic study of 63 cases. Am J Surg Pathol.

[5] Moch H, Amin MB, Berney DM, Compérat EM, Gill AJ, Hartmann A (2022). The 2022 World Health Organization classification of tumours of the urinary system and male genital organs—part a: renal, penile, and testicular tumours. Eur Urol.

[6] Little JS, Foster RS, Ulbright TM, Donohue JP (1994). Unusual neoplasms detected in testis cancer patients undergoing post- chemotherapy retroperitoneal lymphadenectomy. J Urol.

[7] Rice KR, Magers MJ, Beck SDW, Cary KC, Einhorn LH, Ulbright TM (2014). Management of germ cell tumors with somatic type malignancy: Pathological features, prognostic factors and survival outcomes. J Urol.

[8] Spiess PE, Pisters LL, Liu P, Pettaway CA, Kamat AM, Gomez JA (2008). Malignant transformation of testicular teratoma: a chemoresistant phenotype. Urol Oncol Semin Orig Investig.

[9] Ahmed T, Bosl GJ, Hajdu SI (1985). Teratoma with malignant transformation in germ cell tumors in men. Cancer.

[10] Ulbright TM, Loehrer PJ, Roth LM, Einhorn LH, Williams SD, Clark SA (1984). The development of non‐germ cell malignancies within germ cell tumors. A clinicopathologic study of 11 cases. Cancer.

[11] Colecchia M, Necchi A, Paolini B, Nicolai N, Salvioni R (2011). Teratoma with somatic-type malignant components in germ cell tumors of the testis: a clinicopathologic analysis of 40 cases with outcome correlation. Int J Surg Pathol.

[12] Mikuz G, Colecchia M (2012). Teratoma with somatic-type malignant components of the testis. A review and an update. Virchows Arch.

[13] Magers MJ, Kao CS, Cole CD, Rice KR, Foster RS, Einhorn LH (2014). “Somatic-type” malignancies arising from testicular germ cell tumors: a Clinicopathologic study of 124 cases with emphasis on glandular tumors supporting frequent yolk sac tumor origin. Am J Surg Pathol.

[14] Michael H, Ulbright TM, Brodhecker CA (1989). The pluripotential nature of the mesenchyme-like component of yolk sac tumor. Arch Pathol Lab Med.

[15] Ulbright TM, Roth LM (1987). Recent developments in the pathology of germ cell tumors. Semin Diagn Pathol.

[16] Fichtner A, Richter A, Filmar S, Gaisa NT, Schweyer S, Reis H (2021). The detection of isochromosome i(12p) in malignant germ cell tumours and tumours with somatic malignant transformation by the use of quantitative real-time polymerase chain reaction. Histopathology..

[17] Ikeda K, Monden T, Kanoh T, Tsujie M, Izawa H, Haba A (1998). Extraction and analysis of diagnostically useful proteins from formalin- fixed, paraffin-embedded tissue sections. J Histochem Cytochem.

[18] Hughes CS, Moggridge S, Müller T, Sorensen PH, Morin GB, Krijgsveld J (2019). Single-pot, solid-phase-enhanced sample preparation for proteomics experiments. Nat Protoc.

[19] The Cancer Genoma Atlas. TCGA. National Cancer Institute, National Human Genome Research Institute. 2013

[20] Szklarczyk D, Gable AL, Lyon D, Junge A, Wyder S, Huerta-Cepas J (2019). STRING v11: protein–protein association networks with increased coverage, supporting functional discovery in genome-wide experimental datasets. Nucleic Acids Res.

[21] Dennis G, Sherman BT, Hosack DA, Yang J, Gao W, Lane HC (2003). DAVID: database for annotation, visualization, and integrated discovery. Genome Biol.

[22] Hunter JD (2007). Matplotlib: a 2D graphics environment. Comput Sci Eng.

[23] Waskom M. Seaborn: Statistical Data Visualization. Seaborn. 2012.

[24] Oliveros JC. Venny. An interactive tool for comparing lists with Venn’s diagrams. Accessed 8 Jan 2022.

[25] Camaj P, Seeliger H, Ischenko I, Krebs S, Blum H, De Toni EN (2009). EFEMP1 binds the EGF receptor and activates MAPK and Akt pathways in pancreatic carcinoma cells. Biol Chem.

[26] Wyvekens N, Sholl LM, Yang Y, Tran I, Vasudevaraja V, Dickson BC (2022). Molecular correlates of male germ cell tumors with overgrowth of components resembling somatic malignancies. Mod Pathol.

