# Supplementary figures and images for: Characterizing the mutational burden, DNA methylation landscape, and proteome of germ cell tumor-related somatic-type malignancies to identify the tissue-of-origin, mechanisms of therapy resistance, and druggable targets
Source: Br J Cancer. 2023 Sep 19;129(10):1580–9. doi: 10.1038/s41416-023-02425-5 (PMC10645790; doi:10.1038/s41416-023-02425-5)

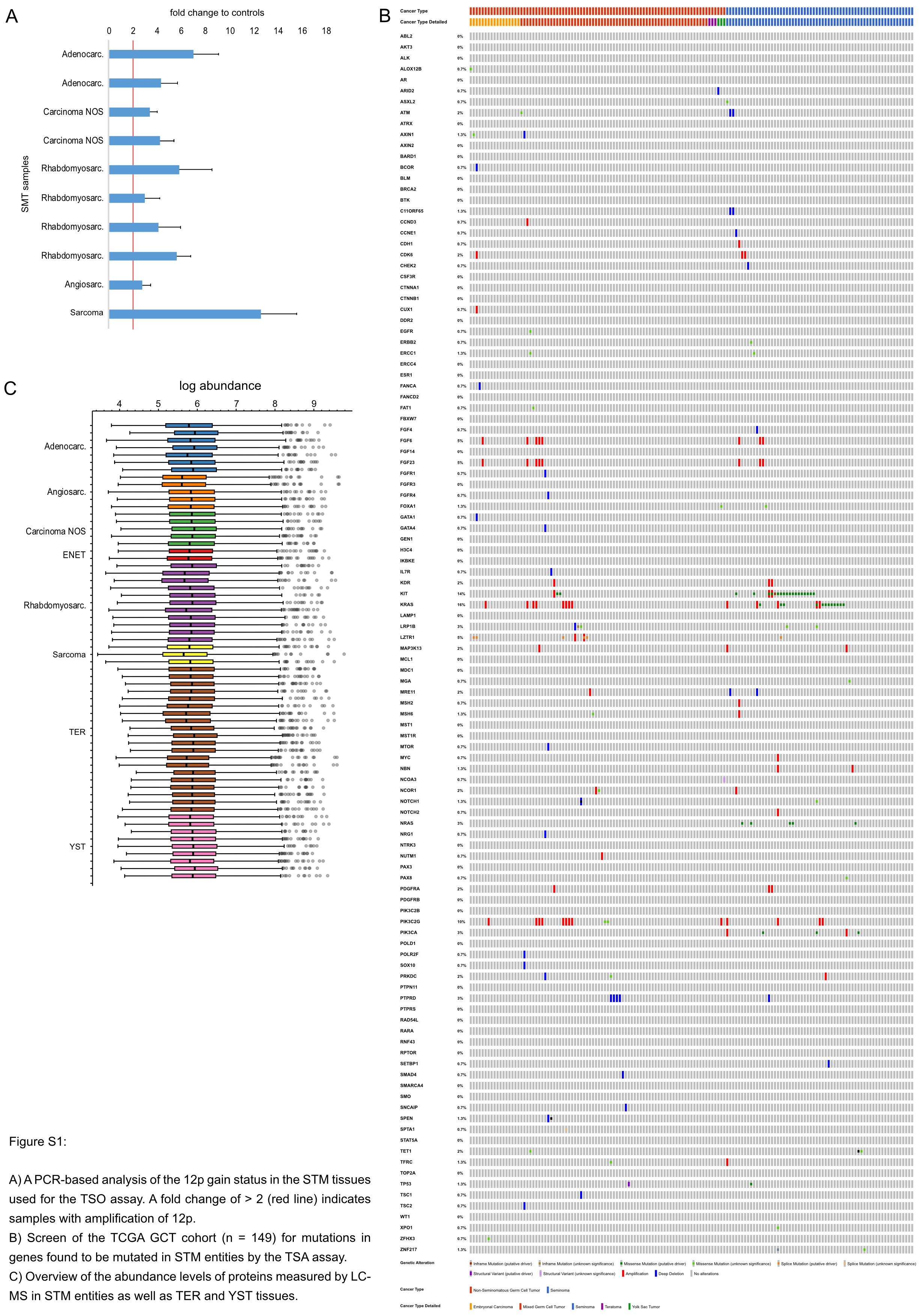

Supplement: Supplementary file 1 — Fig. S1 [file 41416_2023_2425_MOESM1_ESM.tif]

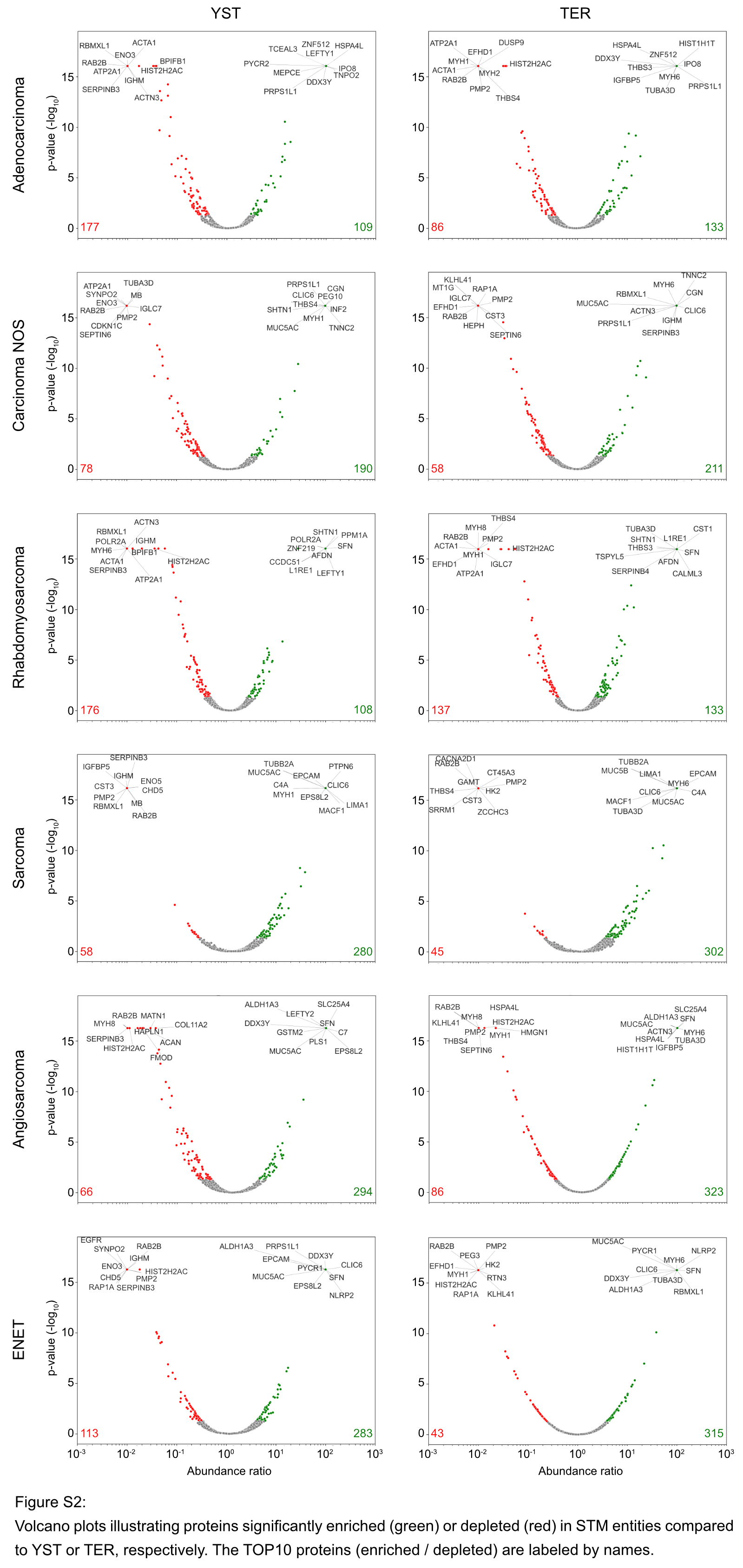

Supplement: Supplementary file 2 — Fig. S2 [file 41416_2023_2425_MOESM2_ESM.tif]

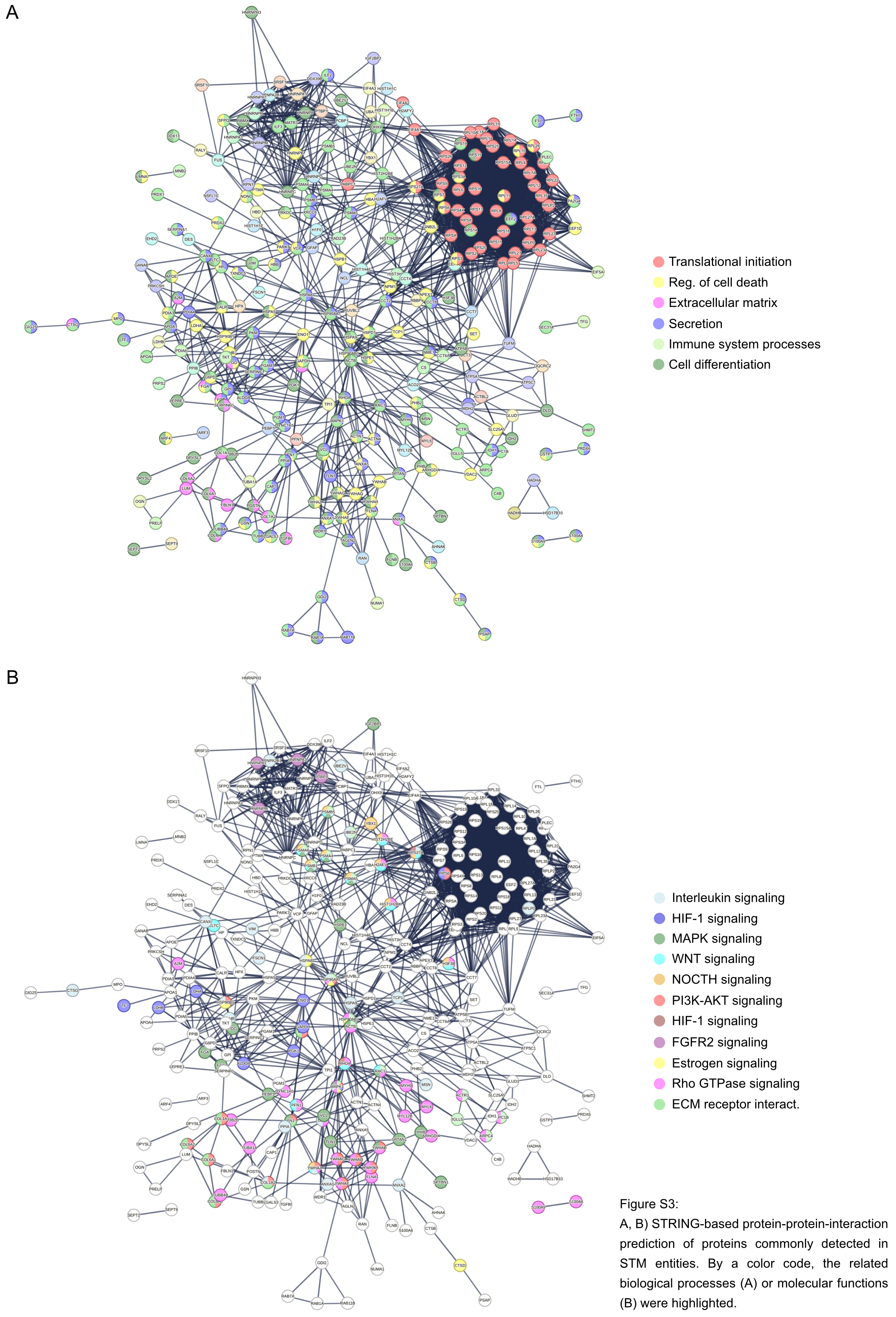

Supplement: Supplementary file 3 — Fig. S3 [file 41416_2023_2425_MOESM3_ESM.tif]

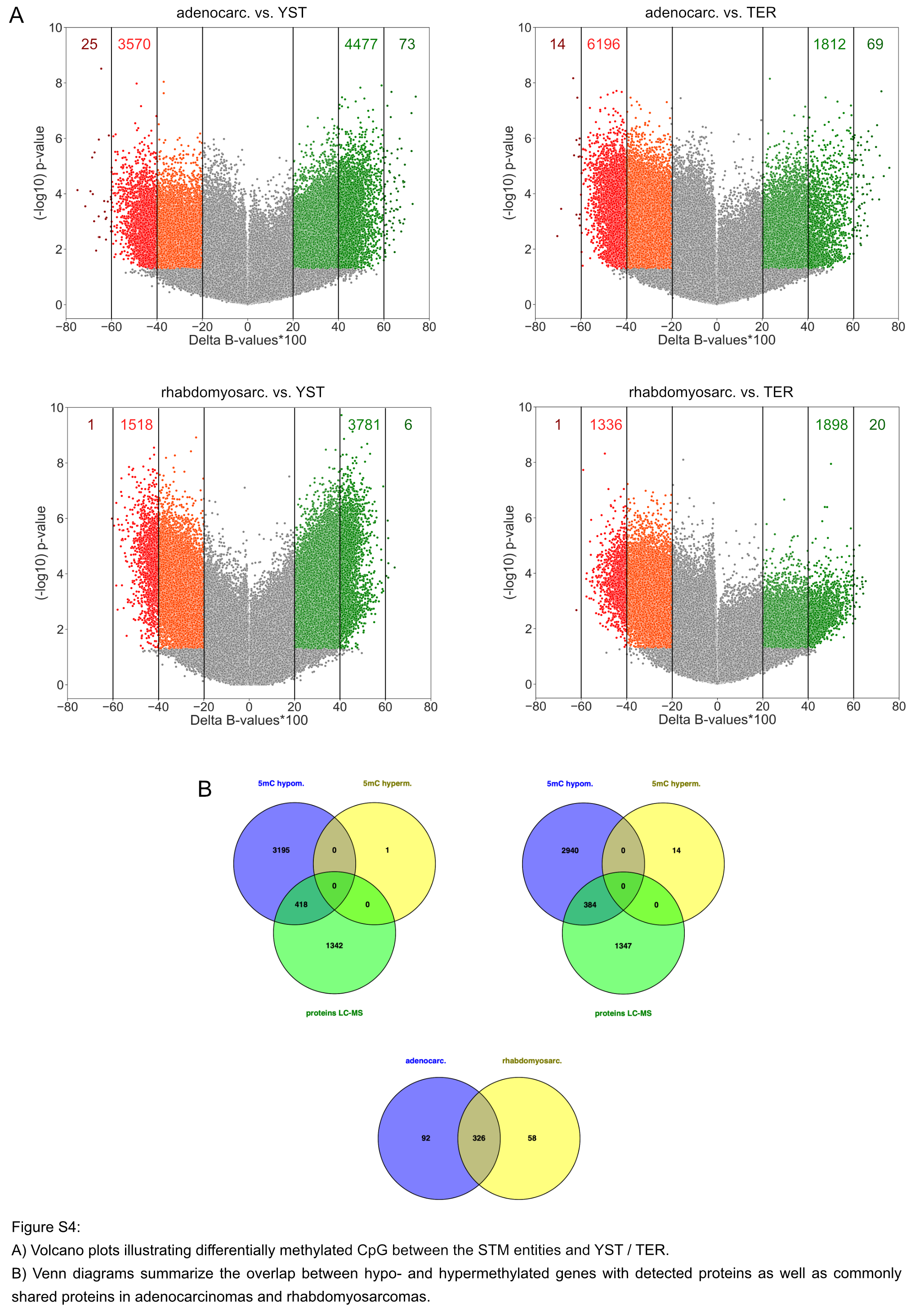

Supplement: Supplementary file 4 — Fig. S4 [file 41416_2023_2425_MOESM4_ESM.tif]
