# Supplementary material for: Characterizing the mutational burden, DNA methylation landscape, and proteome of germ cell tumor-related somatic-type malignancies to identify the tissue-of-origin, mechanisms of therapy resistance, and druggable targets
Source: Br J Cancer. 2023 Sep 19;129(10):1580–9. doi: 10.1038/s41416-023-02425-5 (PMC10645790; doi:10.1038/s41416-023-02425-5)
Supplement: Supplementary file 6 — Supplemental figure and data legends [file 41416_2023_2425_MOESM6_ESM.docx]

**Supplemental figure and data legends:**

Figure S1:

A) A PCR-based analysis of the 12p gain status in the STM tissues used for the TSO assay. A fold change of > 2 (red line) indicates samples with amplification of 12p.

B) Screen of the TCGA GCT cohort (n = 149) for mutations in genes found to be mutated in STM entities by the TSA assay.

C) Overview of the abundance levels of proteins measured by LC-MS in STM entities as well as TER and YST tissues.

Figure S2:

Volcano plots illustrating proteins significantly enriched (green) or depleted (red) in STM entities compared to YST or TER, respectively. The TOP10 proteins (enriched / depleted) are labeled by names.

Figure S3:

A, B) STRING-based protein-protein-interaction prediction of proteins commonly detected in STM entities. By a color code, the related biological processes (A) or molecular functions (B) were highlighted.

Figure S4:

A) Volcano plots illustrating differentially methylated CpG between the STM entities and YST / TER.

B) Venn diagrams summarize the overlap between hypo- and hypermethylated genes with detected proteins as well as commonly shared proteins in adenocarcinomas and rhabdomyosarcomas.

Data S1:

A) Immunohistochemistry data of STM samples.

B, C) TSO array data of analyzed STM samples.

D) LC-MS proteome data.

E) Comparison of proteome data between STM entities and controls.

F) 850k DNA methylation array data.

G, H) List of differentially methylated (< 60 %, > 60 %) CpG between adenocarcinoma (G) or rhabdomyosarcoma (H) compared to YST / TER.

I) Comparison of proteome data (abundance > 1*10^6^) and DNA methylation data (with hypo- (< 20 %) or hypermethylation (> 80 %) in at least 60 % of all questioned CpG linked to an annotated gene).
